# Galectin-3 in Cardiovascular Diseases

**DOI:** 10.3390/ijms21239232

**Published:** 2020-12-03

**Authors:** Valeria Blanda, Umberto Marcello Bracale, Maria Donata Di Taranto, Giuliana Fortunato

**Affiliations:** 1 Dipartimento di Medicina Molecolare e Biotecnologie Mediche, Università degli Studi di Napoli Federico II, 80131 Naples, Italy; valeria.blanda@izssicilia.it (V.B.); fortunat@unina.it (G.F.); 2 Istituto Zooprofilattico Sperimentale della Sicilia, via Gino Marinuzzi 3, 90129 Palermo, Italy; 3 Dipartimento di Sanità Pubblica, Università degli Studi di Napoli Federico II, 80131 Naples, Italy; umbertomarcello.bracale@unina.it; 4 CEINGE S.C.a r.l. Biotecnologie Avanzate, 80131 Naples, Italy

**Keywords:** Galectin-3, atherosclerosis, cardiovascular diseases, biomarker, inflammation

## Abstract

Galectin-3 (Gal-3) is a β-galactoside-binding protein belonging to the lectin family with pleiotropic regulatory activities and several physiological cellular functions, such as cellular growth, proliferation, apoptosis, differentiation, cellular adhesion, and tissue repair. Inflammation, tissue fibrosis and angiogenesis are the main processes in which Gal-3 is involved. It is implicated in the pathogenesis of several diseases, including organ fibrosis, chronic inflammation, cancer, atherosclerosis and other cardiovascular diseases (CVDs). This review aims to explore the connections of Gal-3 with cardiovascular diseases since they represent a major cause of morbidity and mortality. We herein discuss the evidence on the pro-inflammatory role of Gal-3 in the atherogenic process as well as the association with plaque features linked to lesion stability. We report the biological role and molecular mechanisms of Gal-3 in other CVDs, highlighting its involvement in the development of cardiac fibrosis and impaired myocardium remodelling, resulting in heart failure and atrial fibrillation. The role of Gal-3 as a prognostic marker of heart failure is described together with possible diagnostic applications to other CVDs. Finally, we report the tentative use of Gal-3 inhibition as a therapeutic approach to prevent cardiac inflammation and fibrosis.

## 1. Introduction

Cardiovascular diseases (CVDs) are the main cause of morbidity and mortality world-wide [1], constituting 17.9 million deaths in 2016 [2]. Atherosclerosis is responsible for a large proportion of CVDs, including ischaemic heart disease or coronary artery disease, cerebrovascular disease, and diseases of the aorta and other arteries [3]. Over the last decades, cardiovascular mortality rates have declined in many developed countries thanks to a combination of population-wide primary prevention, leading to reduced incidence rates, together with improved individual health-care intervention strategies after cardiovascular events [2]. Despite the decreasing incidence, CVD prevention remains a major challenge to delay or minimize fatal events [4]. Among the panel of candidate molecules for improving CVD assessment, standardized biomarkers include brain natriuretic peptide (BNP) and N-terminal prohormone of BNP (NT-pro-BNP), cardiac troponins, C-reactive protein (CRP) and cardiac enzymes such as creatine kinase (CK), CK-myocardial isoenzyme (MB) [1]. Since 2014, the Food and Drug Administration (FDA) included Galectin-3 (Gal-3) in the list of validated cardiovascular biomarkers [5]. Gal-3 is a β-galactoside-binding protein involved in the regulation of several physiological cellular functions, such as cellular growth, proliferation, apoptosis, differentiation, cellular adhesion, and tissue repair.

This review is focused on the involvement of Gal-3 in cardiovascular diseases and its role in the pathogenesis of atherosclerotic- and non-atherosclerotic-CVDs. The biological role and molecular mechanisms of Gal-3 in CVDs are explored, as well as its role as a diagnostic and prognostic circulating marker of CVDs. Finally, we discuss the possible implication of Gal-3 as a molecular target for new promising therapies for the treatment of CVDs.

### Galectin-3

Galectin-3 (Gal-3) belongs to a lectin family, acting as a galactoside-binding protein involved in many biological processes, such as controlling cell–cell and cell–matrix interactions, adhesion, proliferation, apoptosis, pre-mRNA splicing, immunity and inflammation [6,7].

**Figure 1:**
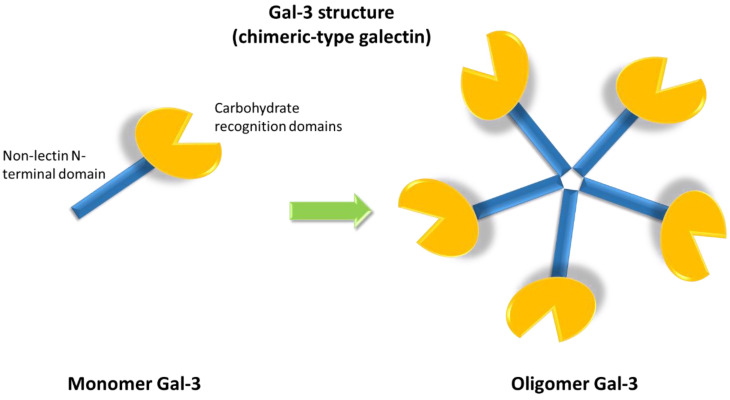
Galectin-3 (Gal-3) chimeric structure characterized by a carbohydrate recognition domain and a non-lectin N-terminal domain that promotes oligomerization, allowing for the formation of pentamers.

The galectin family includes fifteen members, classified into three groups according to their structures: proto, chimera and tandem repeat types. Gal-3 is the only member characterized by a chimeric structure (Figure 1), showing a carbohydrate recognition domain and a non-lectin N-terminal domain promoting oligomerization [8]. Indeed, Gal-3 is the only galectin able to form pentamers. The N-terminal domain of Gal-3 contains a phosphorylation site at serine 6 regulating its nuclear localization and reducing the affinity for its ligands [9,10].

The region between the carbohydrate recognition domains and the N-terminal domain forms a collagen-like sequence of about 100 amino acids and contains the collagenase cleavable H-domain [11].

The protein was originally identified in murine peritoneal macrophages and named MAC-2 because of its capacity to identify a macrophage sub-population [12]. The protein was also identified in different pathways and independently described as IgE binding protein, L-29, CBP30 or CBP35 [13].

In humans, Gal-3 is a 35 kDa protein coded by the *LGALS3* gene located on chromosome 14 [14]. Human Gal-3 is expressed by macrophages during phagocytosis and affects the differentiation and growth of various immune cells, which play a role in angiogenesis. Moreover, Gal-3 is able to induce migration and proliferation of endothelial cells [15].

The protein acts during embryogenesis and is expressed in principal and intercalated cells of the collecting duct of the kidney as well as in later stages of nephrogenesis [16]. Gal-3 is mainly present in the cytoplasm, but it can also be found in the nucleus, on the cell surface and in the extra-cellular environment [17].

The different locations of Gal-3 account for its various functions:

- cytoplasm: regulation of the cell cycle, inducing proliferation and anti-apoptotic effects, through its interaction with survival-associated proteins [14,18,19,20,21,22,23].
- nucleus: regulation of gene transcription and promotion of pre-mRNA splicing [6,22,23].
- cell- surface: involved in lattice assembly, a multi-dimensional organization consisting of Gal-3 in its different forms and several types of saccharide ligands [13,24]. The main functions of lattice are regulation of diffusion, compartmentalization and endocytosis of plasma membrane glycoproteins and glycolipids, selection, activation and arrest of T-cells, signaling of receptor kinase and membrane receptors [25].
- extracellular environment: binding to different cell surface and extracellular matrix glycans, in order to induce cell adhesion, migration, and growth regulation, mainly pro-apoptotic effects [11,26,27,28].

**Figure 2:**
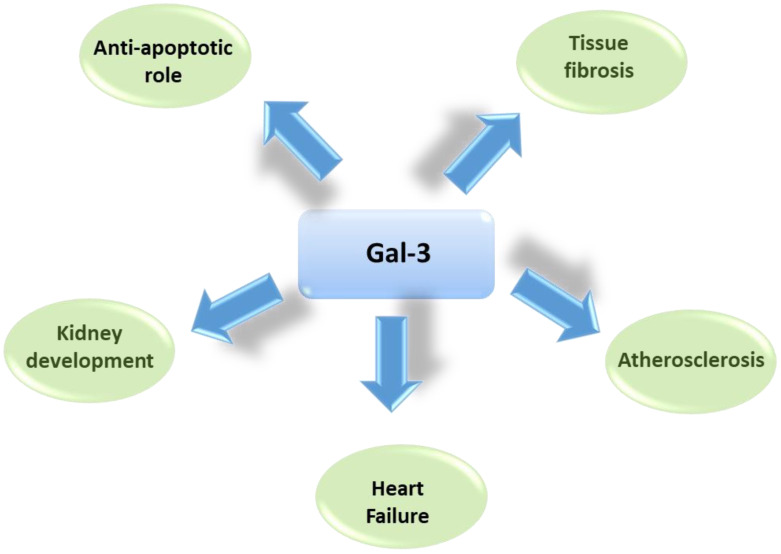
Schematic representation of main roles of Gal-3 in several physiologic and pathogenetic mechanisms.

Due to its multifunctional role, the protein also plays a relevant role in many different clinical conditions and diseases, ranging from asthma to cancer, gastritis, obesity and renal diseases, as reported in Figure 2 and recently reviewed [6].

## 2. Molecular Mechanisms

### 2.1. Gal-3 in Inflammation and Atherosclerosis

As a potent inflammatory protein, Gal-3 contributes to the initiation and amplification of the inflammatory response, being related to both acute and chronic inflammation [29].

In the acute inflammatory response, Gal-3 acts as a promoter of chemoattraction of monocytes/macrophages [30], neutrophil clearance [31], opsonization of apoptotic neutrophils [32], and mast cell degranulation [33]. Moreover, in macrophages and epithelial cells, Gal-3 binds galactoside-associated membrane remnants from vacuoles produced by some intravacuolar bacteria to actively evade the phagolysosomal pathway of the host [34,35,36]. This binding allows to target damaged vesicles for autophagy through the recruitment of autophagy adaptor proteins.

Gal-3 has a wide distribution in primary and secondary lymphoid organs and in circulating immune cells [37]. In addition, Gal-3 is up-regulated in both CD4^+^ and CD8^+^ activated murine T lymphocytes but not in resting T cells. Gal-3 inhibition by an antisense oligonucleotide suppresses the proliferation of T cells [38]. This evidence suggests an active role of Gal-3 in inflammation promotion.

Although inflammation is essential for tissue healing, constant and sustained inflammation may lead to tissue damage and organ failure. Atherosclerosis is a complex inflammatory process initiated by altered cellular permeability of the arterial walls and focal sub-endothelial accumulation of LDL cholesterol (LDL-C), leading to the development of atherosclerotic plaques characterized by inflammation and oxidation [39,40]. The subsequent inflammatory response involves a massive participation of monocytes and macrophages altering the structure of the intima and media layers of the wall [3,41]. Endothelial cells become activated and SMCs actively proliferate, producing extracellular matrix. The narrowing of the artery lumen leads to a decreased blood flow that can be completely arrested in case of a thrombus formation on the plaque surface, causing acute events such as acute myocardial infarction (AMI) or stroke. The involvement of many inflammatory markers in the atherosclerotic process has been investigated over the years [42,43,44,45] and a potential role of Gal-3 as a mediator for atherosclerosis emerged [46].

By studying samples from young adult trauma victims, a higher concentration of Gal-3 was detected in foam cells and macrophages compared to the vascular smooth muscle cells (VSMC) of atherosclerotic lesions [47]. Gal-3 also regulates chronic inflammation at the cardiovascular level, promoting osteogenic differentiation of VSMCs that favours macrocalcifications in atherosclerotic plaques [1,48,49].

Gal-3 was expressed not only by inflammatory cells in unstable plaque regions, but also by VSMCs in fibrosis and particularly of sheet-like/lamellated calcification areas, indicating a more stable pattern [50]. Based on these data, the authors proposed a molecular mechanism by which Gal-3 modulates not only inflammation, but also vascular osteogenesis, with its expression necessary to acquire a complete osteoblast-like phenotype by VSMCs. Another study highlighted that Gal-3 over-expression in VSMCs induced collagen type I increased synthesis [50], supporting its role as a remodelling factor of the vascular wall [51].

In patients with high-grade carotid stenosis, low Gal-3 intra-plaque concentration was correlated with clinically and ultrasonically defined unstable human carotid plaques, suggesting a possible use of Gal-3 in identifying carotid plaques prone to rupture and cerebral embolization [52].

### 2.2. In Vivo Studies

The possibility of carrying out in vivo studies, with the involvement of animal models, provided further insights into the direct role of Gal-3 in the atherosclerotic process [49,53,54,55,56,57,58,59].

Table 1 reports the in vivo studies performed on different animal models. Using the *Apoe* knock-out mice as atherosclerotic-prone animals, a study revealed an increased expression of Gal-3 within the lesion [54]. Most of the studies inhibiting Gal-3 action by knock-out or by a direct inhibitor such as Modified Citrus Pectin (MCP) demonstrated a decreased formation of atherosclerotic lesions but increased lesions with vulnerable features, i.e., lesions with microcalcification, diffused inflammation and low collagen content. Taken together, these results highlight the conflicting aspects of Gal-3 involvement in atherogenesis and its complications.

### 2.3. Gal-3 in Cardiac Fibrosis and Heart Failure

Cardiac fibrosis is a tissue repair mechanism caused by a progressive accumulation of extracellular matrix in response to injury, inflammation or stress. The process leads to an impaired tissue-repair [60], causing tissue and organ scarring, injury, and reduction or loss of function [61,62].

Gal-3 expression is associated with abundant macrophages, increased fibroblast activity, accumulation of extracellular matrix [63] and with collagen production in the myocardium [64]. The protein is also expressed by fibroblasts and macrophages following stressful events [60,65,66].

Gal-3 increase was evidenced in rat models after myocardial infarction, showing a later peak in non-infarcted myocardium involved in cardiac remodelling [67]. Rats intrapericardially infused with Gal-3 reported an enhanced macrophage and mast cell infiltration, increased cardiac interstitial and perivascular fibrosis, and cardiac hypertrophy [68].

After its activation, Gal-3 forms a lattice complex entrapping TGFβ on the cell surface that gives a prolonged signal of fibrotic development [69,70]. These signalling factors, together with mechanical stress, transform quiescent fibroblasts into active collagen-producing myofibroblasts [71].

Gal-3 inhibition by MCP is effective in decreasing both the ischemic area and the fibrotic remodeling in a rat model of ischemia/reperfusion [72]. Similarly, silencing of Gal-3 in cardiomyocytes isolated from rats and treated to simulate ischemia/reperfusion, resulted in a decreased apoptosis thanks to its interaction with bcl-2 [73].

Closely related to the fibrotic process, Heart Failure (HF) is a clinical syndrome caused by a structural/functional cardiac abnormality, resulting in a reduced cardiac output and/or elevated intra-cardiac pressure at rest or during stress, leading to typical symptoms and signs (e.g., breathlessness, fatigue, elevated jugular venous pressure, pulmonary crackles and peripheral oedema) [74]. The two major pathophysiological processes contributing to HF development are inflammation and fibrosis, which affect tissue architecture, electrical conduction, mechano-electrical coupling and force generation by cardiomyocytes [75]. The prevalence of HF is globally increasing, mainly due to population aging [76] and the more successful treatment of cardiovascular diseases preceding HF, such as myocardial infarction, myocarditis and hypertension [77].

Gal-3 is involved in the pathophysiology of HF [78] mainly because of its role in cardiac ventricular remodeling [71,77]. Normally, Gal-3 expression in the heart is low, whereas its synthesis and secretion increase in HF [63,79,80]. Gal-3 initially plays a protective role in the heart through its anti-apoptotic and anti-necrotic functions, while the prolonged expression of this protein leads to fibrosis and adverse remodeling of the damaged tissue [78]. Gal-3 binding sites are mainly localized in the myocardial matrix, fibroblasts and macrophages. At the site of injury, Gal-3 is secreted into the extracellular space and activates resting fibroblasts into matrix-producing fibroblasts. The role of Gal-3 in fibroblast activation involves up-regulation of the cytoskeletal proteins, the synthesis of new matrix components such as type I collagen and the inhibition of extracellular matrix component degradation down-regulating matrix metalloproteinases [78]. Moreover, another study showed that Gal-3 infusion caused myocardial fibrosis, which was neutralized by an anti-fibrotic agent, suggesting that Gal-3 may be involved in the development and resolution of fibrosis [81].

In a rat model of hypertensive HF, recombinant Gal-3 infusion into the pericardial sac for 4 weeks induced excessive collagen deposition and cardiac dysfunction, which is likely to develop into HF [63].

Despite the fact that Gal-3 cardiac levels were increased in hearts from different mouse models of heart disease, Nguyen et al. demonstrated that Gal-3 circulating levels were high in mice with fibrotic cardiomyopathy, whereas normal levels were observed in the dilated cardiomyopathy mouse model [82]. The authors also observed that β-adrenoceptor activation is responsible for increased Gal-3 levels in both cardiac tissue and plasma.

### 2.4. Gal-3 and Other Cardiovscular Diseases

Atrial fibrillation (AF) is the most frequent arrhythmia and it is associated with structural, electrical, genomic and hormonal atrial remodeling [79]. More recently, the concept of fibrotic atrial cardiomyopathy was introduced—defined as a progressive invasion of atrial myocardium by fibrosis, which favors initiation and maintenance of AF [83]. A direct role of Gal-3 in AF was demonstrated by its action in: (i) promoting the retention of cytokine receptors on the atrial myofibroblast membrane, through entrapment within Gal-3 lattice [70]; (ii) interaction of the extracellular pentameric Gal-3 with molecules, such as TGF-β, which could contribute to initiate fibrogenesis [79]; (iii) promoting nuclear translocation of transcription factors such as β-catenin. In the nucleus, β-catenin induces the activation of transcription factors promoting collagen transcription [84].

After an experimentally induced stroke in mice, a high number of Gal-3 positive cells in the brain resulted, which was associated with a smaller infarct size and a better functional outcome, highlighting the contribution of Gal-3 to postischemic brain remodeling [85].

A critical role in venous thrombosis was also reported for Gal-3 and Gal-3–binding protein, which interact with the thrombus–vein wall interface contributing to thrombus formation through proinflammatory, IL-6–dependent mechanisms [86].

A recent paper suggested the involvement of Gal-3 in autoimmune myocarditis; in fact, Gal-3 knock-out mice developed more severe myocarditis and heart hypertrophy than wild-type mice upon induction of experimental autoimmune myocarditis [87].

## 3. Galectin-3 as a Circulating Marker

Biomarkers are objectively measured characteristics that can be used as indicators of normal biological or pathogenic processes [88] and may be useful for improving diagnosis, prognosis and risk stratification of cardiovascular diseases, including some genetic conditions [89,90,91,92,93,94,95]. A recent study established the upper reference limit (URL) of Gal-3 in a population of healthy subjects, identifying age as an important biological variable that affects Gal-3 concentration, suggesting multiple diagnostic cut-offs related to the different age groups [96].

To date, Gal-3 measurement is recommended by the 2017 Guidelines of the American Heart Association for risk stratification and prognosis evaluation of patients with HF [97]. Besides this application, Gal-3 levels have been investigated as biomarkers of several CVDs, in particular, in cardiovascular diseases initiated and stimulated by inflammation [98]. Recently, a possible role of Gal-3 as a marker of therapy-induced cardiotoxicity was also investigated; however, no association was found [99].

### 3.1. Atherosclerotic Plaques

Several studies evaluated the role of Gal-3 plasma levels as biomarkers of atherosclerosis highlighting correlations with plaque instability. For example, a study on patients with coronary artery disease (CAD) showed that unstable patients had higher plasma Gal-3 levels compared to the stable ones [100]. Moreover, the authors found a trend of correlations between plasma Gal-3 levels and the number of compromised vessels. This study further validates the association of Gal-3 with macrophage activation and monocyte attraction, thereby supporting its possible use as a biomarker of atherosclerotic plaques and of their destabilization.

Hypercholesterolemia is a well-known risk factor for atherosclerosis development, and the most severe form, the homozygous familial hypercholesterolemia, is associated with very early CVDs [101,102]. Despite being invasive, lipoprotein apheresis is one of most effective treatments of severe hypercholesterolemia and has been shown to contribute to the decrease in circulating Gal-3 levels in humans, enhancing the benefits from this treatment [103].

High levels of Gal-3 have been associated with high-grade carotid atherosclerosis [104,105]. In the Atherosclerosis Risk in Communities (ARIC) study, patients in the fifth quintile according to Gal-3 concentration showed a higher cIMT in comparison with patients in the first quintile [104]. Our research group recently demonstrated that high plasma concentrations of Gal-3 are reliable biomarkers of advanced atherosclerotic plaque as they are associated with plaque presence independent of age, sex, LDL-cholesterol levels and a previous AMI [105].

### 3.2. Atherosclerotic Cardiovascular Diseases

Gal-3 concentration and carotid intima-media thickness (cIMT) values emerged as independent predictive indicators of mortality risk in patients after MI during mid-term follow up [106].

Among a large population of 5805 elderly (mean age 69 years), low Gal-3 levels were a good negative risk marker for cardiovascular events, being associated with a low incidence of CVD events at a short-term follow-up. Interestingly, for risk assessment Gal-3 levels are even more relevant than the absence of carotid plaque evaluated by ultrasound [107].

As to patients undergoing coronary angiography for CAD, during long-term follow-up, cardiovascular deaths occurred more commonly in the high Gal-3 tertile, suggesting that plasma Gal-3 level is an independent predictor of cardiovascular mortality in patients with CAD [108]. In addition, a positive correlation between serum Gal-3 levels and CAD severity was also reported in another study [109].

In patients with CAD after AMI, a significant decrease in Gal-3 concentration was observed in subjects without acute events during follow-up observation [110].

High Gal-3 levels measured at the time of incident AMI were found to be an independent predictor of mortality, suggesting that measures of Gal-3 may be used for risk stratification post-AMI [111]. Furthermore, in post-myocardial infarction patients, it was shown that plasma Galectin-3 levels predict a high risk of deleterious vascular dysfunctions at 6 months [112].

A recent study highlighted that the association of high Gal-3 levels with cardiovascular events could be influenced by the presence of type-2 diabetes. In fact, Gal-3 plasma levels were associated with cardiovascular events in CAD patients with diabetes, whereas NT-proBNP in patients without diabetes [113].

A significant prognostic value of serum Gal-3 has also been reported for predicting severe abdominal aortic calcification in patients with end-stage renal disease undergoing hemodialysis [114].

High serum Gal-3 levels were associated with the presence of acute ischemic stroke (AIS) and are correlated with AIS severity and infarction volume [115]. Moreover, serum Gal-3 levels were significantly higher in patients with a poor outcome. Similarly, higher Gal-3 serum levels were found in patients with large artery atherosclerotic stroke compared to controls. In addition, an independent association was evidenced between Gal-3 levels and unfavorable outcomes after ischaemic stroke [116].

The increase in Gal-3 plasma levels in patients who underwent carotid endarterectomy were also associated with increased incidence of postoperative stroke [117]. In particular, in women, Gal-3 was a predictor of postoperative stroke even after correction for traditional risk factors, suggesting a potential role of the protein as a marker to predict postoperative cerebrovascular ischemic events.

As to atherosclerosis in other artery regions, a different distribution of Gal-3 was observed in normal arteries and arteries from patients with PAD. Indeed, Gal-3 was expressed in the adventitia in normal arteries, while in the arteries of PAD patients, Gal-3 was expressed mainly in the media and, to a lesser amount, in the adventitia and the intima. Moreover, serum Gal-3 levels were higher in PAD patients than in the control group, correlating well with other circulating markers of inflammation or oxidative stress [118].

Gal-3 concentrations were significantly associated with increased cardiovascular mortality risk in patients with peripheral artery disease (PAD) followed-up for 5 years [119].

Recently, higher levels of Gal-3 measured in 9851 ARIC Study participants free of PAD at baseline were significantly associated with an elevated risk of PAD and critical limb ischemia over 17.4 years of follow-up. This association was independent of traditional risk factors, including levels of C-reactive protein levels, suggesting that Gal-3 may enhance the prediction of incident PAD [120].

### 3.3. Heart Failure

The Gal-3 serum level has been approved as a diagnostic marker for risk stratification and prognosis evaluation of HF patients according to the 2017 ACC/AHA/HFSA Guidelines for the Management of HF [121]. Although the role of BNP and NT-pro-BNP for HF diagnosis and prognosis is indisputable, Gal-3 levels can improve patient management, providing additional information about possible risks of further hospitalization and death. A possible use of Gal-3 as a biomarker in HF was widely investigated and recently reviewed [77,122].

The threshold of 17.8 ng/mL of Gal-3 serum levels is usually considered to discriminate between HF patients at low and high risk for clinical complications [123]. Based on this cut-off, a pooled data analysis carried out in patients with HF (n = 902) enrolled in three cohorts (COACH, PRIDE and UMD H-23258) showed that plasma Gal-3 concentrations can be useful in HF hospitalized patients for near-term rehospitalization prediction [124].

The Valsartan Heart Failure Trial 2013 (n = 1650) reported that Gal-3 increase in patients with symptomatic HF was independently associated with worse outcomes [125]. A meta-analysis of 18 studies, involving 32,350 participants among the general population and patients with HF found a correlation between elevated plasma Gal-3 and risk of all-cause mortality, CVD mortality, and HF [126]. Conversely, a study performed in 1161 patients enrolled in the RELAX-AHF trial did not find an independent association of Gal-3 serum levels with cardiovascular mortality within 180 days of hospitalization for acute HF [127]. A recently published meta-analysis evaluated the prognostic role of Gal-3 serum levels in 7057 patients with acute HF [128], confirming that higher serum levels may be associated with poor prognosis in such patients.

Gal-3 may provide additional information in HF prognosis and risk stratification since the combination of biomarkers could be more informative than single biomarkers, as reported in the Scientific Statement from the American Heart Association [97]. The additional prognostic value of Gal-3 was demonstrated in the HF-ACTION study on a cohort of about 900 ambulatory patients with HF [129]. In this study, patients with high levels of both NT-proBNP and Gal-3 showed a Hazard Ratio of 2.19 for hospitalization at 4 years of follow-up compared to patients with low levels of both markers. In addition, a modest correlation of Gal-3 and NT-proBNP levels was observed, suggesting that these two markers usually increase simultaneously. However, in this study, the prognostic role of Gal-3 levels turns out to be inconsistent in a multivariate analysis including NT-proBNP levels, probably as a consequence of the above-mentioned correlation. Other studies demonstrated that the predictive value of Gal-3 is independent of NT-proBNP levels, age, gender and estimated glomerular filtration rate (eGFR) [130]. Decreased eGFR values are an additional risk factor in HF patients, often leading to re-hospitalization. Unfortunately, natriuretic peptide levels are increased in patients with low eGFR independently of the presence of HF. A recent review highlighted the different biomarkers that can be used for HF prognosis in patients with chronic kidney disease, indicating that Gal-3 could be more useful than NT-proBNP being less influenced by eGFR [131]. In patients with chronic kidney disease without signs of HF at recruitment, high Gal-3 levels were associated with early symptomatic changes of HF, although with a hazard ratio lower than that of growth differentiation factor-15 (GDF-15) [132].

The study of a large population of subjects free of HF (22,756 participants) followed for more than 12 years revealed a moderate contribution of Gal-3 in the prediction of HF development only in women [133]. In addition, differently from NT-proBNP, Gal-3 circulating levels were not associated with measures of cardiac mechanics in elderly subjects, indicating that Gal-3 cannot be used as a biomarker of pre-clinical heart failure [134]. On the other hand, in a community-based cohort, elevated plasma levels measured at midlife of Gal-3 are not only associated with incident heart failure, but also with incident coronary heart disease, ischemic stroke, and total mortality [135].

Taken together, this evidence highlights a prominent role of Gal-3 in the prognosis of HF patients, but a conflicting role in terms of prediction or early diagnosis of HF.

#### Repeated Measurements of Gal-3 Levels

While a single measurement of Gal-3 levels is informative, the main characteristic that justifies the use of Gal-3 for HF management is the lower biological variability compared to other cardiac markers, with the within-subject variability being equal to 8.1% in both healthy subjects and HF patients [136].

Since the low within-subject variability justifies the usefulness of repeated measurements, Gal-3 levels have been evaluated in patients with HF enrolled in the CORONA (n = 1329) and COACH (n = 324) trials, with repeated measurements at baseline and at 3 or 6 months, respectively. An increase of Gal-3 by >15% indicated a 50% higher relative hazard of adverse events (hospitalization and mortality) also after correction for age, sex, diabetes mellitus, left ventricular ejection fraction, renal function, medication, suggesting a significant prognostic value of repeated Gal-3 measurements [137]. As to the general population, in the longitudinal study carried out on 2477 participants in the Framingham Heart Study Offspring cohort, increases in Gal-3 levels during the observation time were related to future HF, CVD and all-cause mortality [138].

## 4. Gal-3 and Statins

In recent years, several studies have been carried out to investigate the regulation of Gal-3 levels by statin therapy, as well as the association of Gal-3 serum levels with the rate of cardiovascular events after statin therapy.

In a model of 36-week-old *Apoe*^−/−^ mice fed a high-cholesterol diet for 5 days, the intraplaque Gal-3 levels were correlated with plaque inflammation. In this mouse model, the statin treatment markedly reduced both intraplaque Gal-3 levels and macrophage content compared to their saline-treated counterparts [54]. This study suggested the possibility of using Gal-3 as an indicator of plaque stabilization with statin therapy.

A recent study demonstrated an association of Gal-3-negative macrophages accumulation with atherosclerotic plaque development in *Apoe*^−/−^ mice. This study also revealed that statin therapy induced plaque regression together with decreased accumulation of Gal-3-negative macrophages. These findings suggest that statins act by stimulating the protective role of Gal-3 in regulating macrophage polarization and in delaying plaque progression [59].

As for humans, the impact of statin therapy on Gal-3 levels in 78 consecutive patients (40 symptomatic, 38 asymptomatic) undergoing carotid endarterectomy has been investigated [52]. The authors showed an association between long-term statin treatment and elevated Gal-3 levels and reduced macrophage intra-plaque concentrations (as an indicator of plaque stability) compared to patients with short-term treatment. The study supports the hypothesis that long-term statin treatment induces an increase in the intra-plaque Gal-3 concentration, mediating plaque stabilization.

Although these two studies have been performed in mice and humans, respectively, they suggest two opposite regulations of Gal-3 levels by statins. Additional studies are required to disclose the real Gal-3 regulation.

An interesting study [139] was conducted on 1492 patients with ischaemic systolic HF enrolled in the CORONA study. These patients were randomly assigned to 10 mg/day of rosuvastatin or placebo, and Gal-3 was measured in the plasma. Patients with Gal-3 values < 19.0 ng/mL treated with rosuvastatin showed a decreased risk of the primary endpoint (i.e., cardiovascular death, myocardial infarction, or stroke), lower total mortality and lower rate of the combined endpoint of total mortality and hospitalization for worsening HF compared to patients with Gal-3 > 19.0 ng/mL.

A recent review discussed the evidence surrounding a possible use of Gal-3 for the prediction of statin-therapy outcomes and suggested that low Gal-3 levels could help to identify a subset of subjects with a more functional myocardium [140].

## 5. Gal-3 as a Therapeutic Target for CVD

Since the pathogenic role of Gal-3 in CVD has been established, several hypotheses on its therapeutic use have been put forward [14,141].

As an example, the inhibitor MCP, an oligosaccharide belonging to the pectin family and present in the peels of fruits and vegetables, was reported to inhibit Gal-3 by binding to its Carbohydrate Recognition Domain [142]. In rodents showing LV diastolic dysfunction treated with aldosterone and salt, Gal-3 inhibition through MCP was able to reduce aldosterone-induced cardiac and renal fibrosis and to improve cardio-renal dysfunction [143].

In a murine model of HF with cardiac hyperaldosteronism, the use of MCP prevented the development of myocardial fibrosis and the combination of this molecule and mineralocorticoid receptor antagonists resulted in enhanced effects on cardiac inflammation and fibrosis, by reducing the development of myocardial inflammation and fibrogenesis [144]. On the other hand, treatment with mineralocorticoid receptor antagonists showed a down-regulation of Gal-3 expression in an experimental model of LV systolic dysfunction after AMI and such regulation correlated with lower expression levels of fibrosis [145].

Moreover, MCP turned out to be effective for treating atherosclerosis in mice. In fact, in high-cholesterol diet fed *ApoE*^−/−^ mice, a well-characterized murine model of atherosclerosis, Gal-3 deletion as well as MCP oral administration reduced plaque volume [55].

## 6. Conclusions

Gal-3 is a fascinating protein with a pleiotropic role in several physiologic pathways. The protein is involved in a high number of pathogenetic mechanisms. In cardiovascular diseases, Gal-3 is an active player in fibrosis development, in the atherosclerotic process and more broadly in inflammation-based diseases. The most prominent aspect of this molecule is that it can be successfully used as a risk marker of CVDs if measured in plasma.

Gal-3 measurement should be recommended for prognosis evaluation of HF patients, as suggested by the international guidelines, whereas the prediction value of HF development remains doubtful.

In particular, for HF, it should be remembered that a multi-marker approach is the most useful approach and that measurement of Gal-3 should be repeated during lifetime follow-up because level changes could provide additional risk information.

Gal-3 inhibitors for therapeutic purposes are innovative and interesting perspectives, although further clinical investigations are needed to clarify their potential for CVD prevention.

## Figures and Tables

**Table 1:** Main in vivo studies on the direct role of Gal-3 in the atherosclerotic process.

Papers	Experimental Model	Effects
Menini et al., 2013 [49]	*Lgals3* ^−/−^	No osteogenic differentiation of vascular smooth muscle cells resulting in ↓ plaque stability
Iacobini et al., 2009 [53]	*Lgals3*^−/−^ mice	↑ Lesion area and length; complex lesions and extensive inflammation at the aortic sinus.
Lee et al., 2013 [54]	*Apoe*^−/−^ mice	↑ Gal-3 expression within atherosclerotic plaques proportionally to the extent of plaque inflammation; Gal-3 colocalization with plaque macrophages’ distribution.
Lu et al., 2017 [55]	*Apoe*^−/−^ mice + inhibitor of Gal-3 (MCP)	↓ size of atherosclerotic lesions, ↓number of macrophages and smooth muscle cells in the lesions, ↓endothelial injury.
Nachtigal et al., 2008 [56]	*Lgals3*^−/−^:*Apoe*^−/−^ mice	↓ Atherosclerotic lesions ↓ perivascular inflammatory infiltrates and mast cells.
MacKinnon et al., 2013 [57]	*Lgals3*^−/−^:*Apoe*^−/−^ mice	↓ Atherosclerotic lesions.
Sun et al., 2019 [58]	*Apoe*^−/−^ mice + Gal-3 silencing	↑ microcalcification in the plaque.
Di Gregoli et al., 2020 [59]	*Lgals3*^−/−^:*Apoe*^−/−^ mice	Altered plaque composition, ↓ collagen content, ↑ necrotic area, ↑ invasive capacity of macrophages, ↑ expression of proinflammatory genes.
	*Mmp12*^−/−^:*Apoe*^−/−^ mice	↓ plasma levels of soluble Gal-3; ↑ Gal-3 expression within plaques.
